# GC-MS/MS and LC-MS/MS Identification of Opium and Tropane Alkaloids in Pottery from Funnel Beaker Culture Sites in South-Eastern Poland

**DOI:** 10.3390/ma18235316

**Published:** 2025-11-25

**Authors:** Marcin Osak, Grzegorz Buszewicz, Halina Taras, Anna Zakościelna, Grzegorz Teresiński, Robert Karpiński, Jacek Baj

**Affiliations:** 1 Chair and Department of Forensic Medicine, Medical University of Lublin, Jaczewskiego 8b Street, 20-090 Lublin, Poland; 2 Institute of Archeology, Maria Curie-Skłodowska University in Lublin, 4 M. Curie-Skłodowska sq., 20-031 Lublin, Poland; 3 Department of Machine Design and Mechatronics, Faculty of Mechanical Engineering, Lublin University of Technology, Nadbystrzycka 36 Street, 20-618 Lublin, Poland; 4 Institute of Medical Sciences, The John Paul II Catholic University of Lublin, Konstantynów 1H Street, 20-708 Lublin, Poland; 5 Department of Normal, Clinical, and Imaging Anatomy, Medical University of Lublin, Jaczewskiego 4 Street, 20-090 Lublin, Poland

**Keywords:** GC-MS/MS, LS-MS/MS, Funnel Beaker Culture, vessels, pottery, psychoactive substances, opium, tropane, alkaloids

## Abstract

This study examines the occurrence of psychoactive opium and tropane alkaloids in ceramic vessels from the Funnel Beaker Culture (FBC) using optimized GC-MS/MS and LC-MS/MS analytical protocols. Twelve vessels and potsherds, including miniature forms and collared flasks, were subjected to standardized extraction, derivatization, and chromatographic analysis. The GC-MS/MS method enabled highly sensitive detection of target compounds (LOD < 0.5 ng/g), while LC-MS/MS provided complementary confirmation of analytes. Papaverine was identified in three artifacts (N1, N2, G2) using GC-MS/MS, and its presence was independently confirmed in two of these vessels (N1, N2) by LC-MS/MS. In one miniature vessel (D1), trace amounts of tropane alkaloids—scopolamine and presumptive atropine—were detected following derivatization. Recovery values ranged from 55% to 61%, demonstrating effective alkaloid isolation despite extensive degradation processes and strong interactions between organic residues and the ceramic matrix. The analytical results indicate that psychoactive plant derivatives could have been intentionally used and stored in selected FBC vessels, supporting interpretations related to ritual or medicinal practices. The validated procedures developed in this work provide a robust framework for the detection of unstable biomolecular residues in prehistoric ceramics and can be applied to future archaeometric and materials-science research.

## 1. Introduction

Nowadays archeology constitutes a field of study which by the usage of a range of advanced study techniques, provides a broad insight into the lives of ancient cultures. The interpretation of specific functions and applications of millennia-old objects is a customary demanding task in the field of archeology, requiring interdisciplinary cooperation of various research groups utilizing sophisticated methodologies [1,2]. Formerly archeological studies were based mainly on comparative observation and underlining the visible features such as shape, size, and style of decorations of the vessels. Those previous approaches regularly met with difficulties in the interpretation while relating the obtained data to the function of the discovered item [2,3]. The development of the analytical physical and chemical methods revolutionized the field of archeology, providing, for example, the possibility of classifying exceptionally old objects through data on preserved invisible traces in artifacts [4]. Subsequent studies on vessel organic residues presented the gradual progression of the method’s effectiveness along with the new avenues for biomolecule identification and upscaling their range possible to detect at trace levels. Thus, the answers appeared to previously intractable questions across the themes of vessel functions, human activities concerning food preparation, consumption and exchange, rituals, as well as the interactions between local communities through time and space [2,4,5]. It was recognized that organic residues are commonly present in the embedded surface remains or as an absorbed form within the unglazed porous surface of the vessels. Organic remains may be present as both, visible or invisible amorphous traces and deposited on the inner or outer part of the vessel [6]. According to the literature, most analytical studies conducted so far have focused on lipids as archeological biomarkers, because of their durability and widespread in pottery. Lipids are also highly hydrophobic which limits their loss by solubilization and percolation to the environment. The aforementioned facts provide a wide range of opportunities for lipids determination in pottery, by using contemporary analytical methods [4,6]. The molecular characterization of the preserved lipids (waxes, resins, fats) poses a useful tool for researchers by unraveling questions about the type of commodities stored and consumed along with the ways of their processing, particularly in the case of foodstuff containers [2,6,7]. The main benefit of lipids identification is the ability to distinguish the origin of residues between either plant or animal kingdom. Such indicators of source are for instance plant phytosterols (sitosterol, stigmasterol, campesterol) or animal zoosterols (cholesterol, cholestanol, lanosterol) along with their derivatives [7]. However, some inquiries remain open in terms of an accurate identification of stored commodities which could be of great importance in studies on the unusual uses of vessels, including ritual, ceremonial, or even ascertaining the poisons, medicines, or cosmetics containers [8]. Therefore, there is a need to employ more selective biomarkers for instance plant alkaloids, which are generally attributed to specific species or taxa and featured by various physiological or toxic actions to animal or human organisms [8,9]. However, unlike lipids, alkaloids are generally much more prone to degradation in ancient objects or cultural layers, where numerous environmental factors, such as humidity, pH, microbial processes, long time of residence, or temperature significantly affect their stability [1,6,10,11]. Moreover, the issue of contamination is sometimes encountered, additionally confounding attempts to infer the actual purpose of the vessels. Artificial substances may come from a variety of sources including soil microorganisms transformations, diverse uses of the item over its use life, absorption of groundwater constituents, contamination during human activities about pottery handling, starting from excavation through cleaning, and ultimately analysis [1]. Consequently, a direct determination of alkaloids in pottery with archeological timescales kept in mind poses a truly daunting task. The studies on such molecules depend on stability consideration and the proper selection of durable biomarkers for the target. As an alternative solution, it is potentially useful to analyze the decay products formed from original molecules in the complicated conditions of a prehistoric grave or object of the settlement [2,10].

Funnel Beaker Culture was an archeological culture in north-central Europe that introduced husbandry and farming using pottery daily. Further, the Funnel Beaker Culture is known for presenting the earliest evidence of the introduction of wheeled vehicles in Central Europe. The name of the culture comes from the ceramic vessels that were characteristic of this culture [12].

Although direct evidence of ritual or medicinal practices within the Funnel Beaker Culture (FBC) is limited, archaeological findings suggest that this community engaged in behaviors that could be associated with symbolic or ceremonial activities. The presence of miniature vessels and collared flasks—forms often interpreted as non-culinary—together with their frequent occurrence in funerary contexts, indicates that certain ceramic containers may have been used for handling special substances or participating in ritualized actions. Additionally, archaeobotanical studies document the cultivation and availability of plant species capable of producing psychoactive alkaloids, creating a plausible cultural and environmental framework for their use. These observations provide a reasonable archaeological basis for investigating the presence of alkaloid residues in FBC pottery and support the research hypothesis explored in this study.

**Figure 1:**
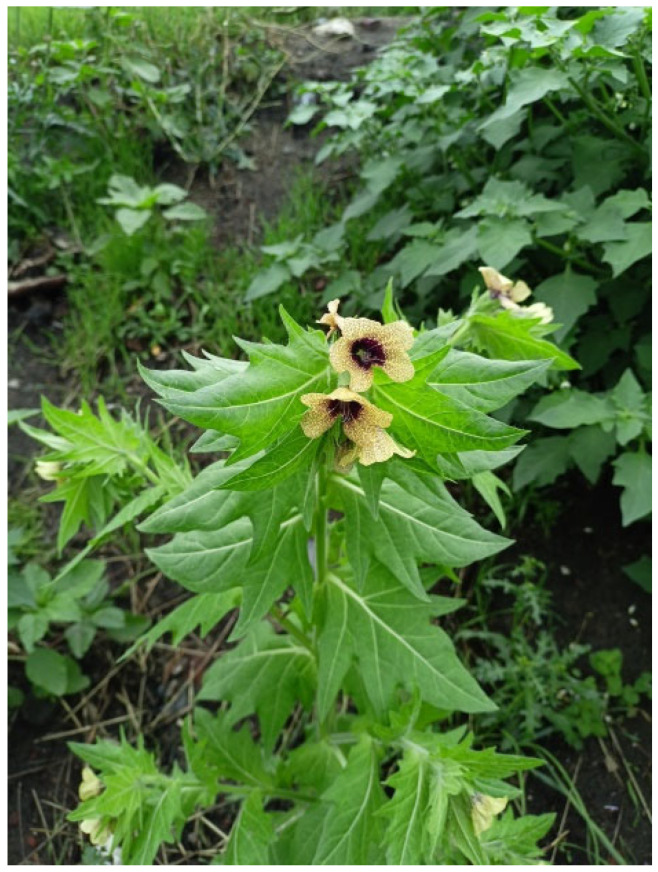
An example of indigenous Polish flora plants of tropane-producing solanaceous, the *Hyoscyamus niger* (Photography by M. Osak).

Despite the difficult challenge, in the following paper, we paid attention to tropane and opium alkaloids, which have psychoactive effects on humans and occur in plant species native to considered area and temporal boundaries (Figure 1), assuming they were very likely used by ancient societies for the ritual purposes [3,8,11].

In our study, we address the issue of inferring the functions of pottery, by utilization of gas chromatography-tandem mass spectrometry (GC-MS/MS) and liquid chromatography-tandem mass spectrometry (LC-MS/MS). The researched ceramic artifacts come from the Funnel Beaker Culture (FBC) sites in eastern Poland. We conducted analyses to determine whether the consumption of psychoactive plants was practiced during the culture under study. The primary task was to identify chemical substances in clay vessels related to ceremonial activities, such as collared flasks and miniature forms.

These preliminary results have already been published in a short paper that focused primarily on the archaeological aspects of the project [13]. The present article provides the first comprehensive and original presentation of the research methodology employed.

## 2. Methods

The methodological approach of this study was designed to ensure reliable detection of psychoactive alkaloids preserved in prehistoric pottery. To achieve this, a representative assemblage of Funnel Beaker Culture vessels and sherds was selected and subjected to carefully controlled laboratory procedures. The workflow included the preparation of archeological samples, application of internal standards, optimized extraction protocols, and comparative chromatographic analyses (GC-MS/MS and LC-MS/MS). This integrated design allowed both the isolation and confirmation of trace organic residues while minimizing the risk of contamination, providing a solid analytical basis for interpreting the potential ritual or medicinal use of the examined artifacts.

### 2.1. Materials

**Figure 2:**
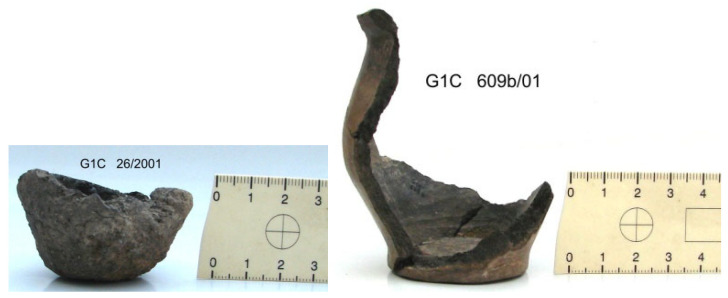
Miniature forms of FBC vessels (**left** G2 and **right** G3), deriving from settlement context in Gródek (Photography by S. Oliwiak, The Museum Zamojskie in Zamość, Poland).

**Figure 3:**
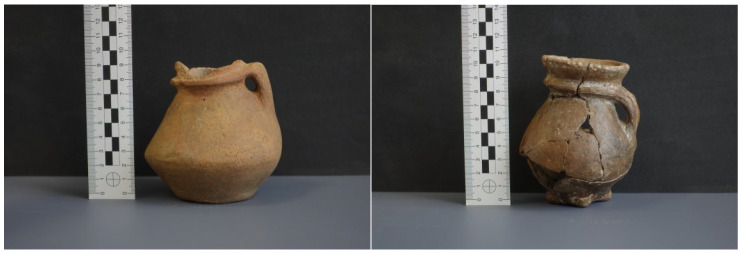
Collared flasks of FBC (**left** N1 and **right** N2), derived from the sites of Nałęczów Plateau (Photography by M. Osak).

The material subjected to our study were complete vessels or their fragments (Figure 2 and Figure 3) of FBC, which were discovered and classified following archeological field research.

Selected: nine specimens of miniature vessels (complete and fragments) from two settlements of the south-eastern group of the FBC—six from Dubeczno 1 (samples D1–D6) [j] and three from Gródek 1C (samples G1–G3) [k], as well as three collared flasks. Two specimens had been discovered in the tombs of the FBC on the Nałęczów Plateau, explored in Wąwolnica 7 (sample W1) [l] and Zgórzyńskie (sample N2—unpublished), while the third flask is a stray find discovered at an unspecified localization, also within the Nałęczów Plateau (sample N1—unpublished).

**Figure 4:**
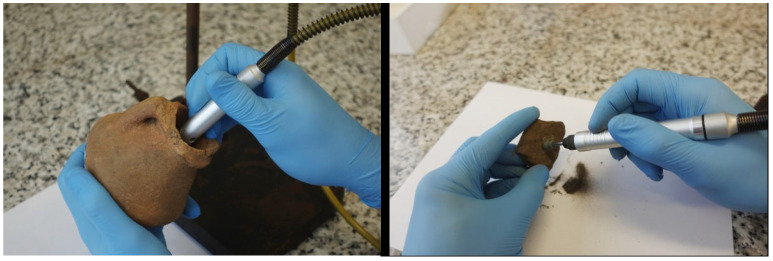
Sampling of interior surface remains of the vessel (**left**), and the potsherd (**right**).

The reference material posed unidentifiable, small clay potsherds, which didn’t show any analytical signal corresponding to targeted substances during our tests. Artificial samples were prepared from reference pottery sherds with standard addition and they served to establish the final analytical conditions (Figure 4).

All of the materials were delivered from the Institute of Archeology of the University of Maria Currie-Skłodowska in Lublin, The Museum of the Chełm Land in Chełm or The Museum Zamojskie in Zamość. The obtained material assemblage was analyzed in the toxicological laboratories of the Chair and Department of Forensic Medicine at the Medical University in Lublin. We provide a description of each object in relation to the archeological origin in Table 1.

### 2.2. Chemicals

The analytical standards of atropine, scopolamine, morphine, codeine and papaverine were purchased from Sigma-Aldrich^®^ (St. Louis, MO, USA) the morphine-D6 and fentanyl from LGC Group^®^ (Teddington, GBR), the acetonitrile, ethyl acetate, methanol and water CHROMASOLV^TM^ LC-MS from Honeywell^®^ (Morris Plains, NJ, USA), the ammonium hydroxide solution ACS reagent, 28.0–30.0% NH_3_ basis from Sigma-Aldrich^®^ (St. Louis, MO, USA), the formic acid from Fluka^®^ (Darmstadt, DEU), the derivatization reagent Sylon BFT (BSTFA with TMCS, 99:1) from Sigma-Aldrich^®^ (St. Louis, MO, USA) and ammonium carbonate ACS reagent from Supelco^®^ (Bellefonte, PA, USA), and the centrifuge vials with tube filters 0.2 µm PTFE from Whatman^®^ (Maidstone, GBR).

### 2.3. Preparation of Pottery Samples

Stock solutions of each alkaloid were prepared in methanol as a medium and further diluted for particular calibration levels. The pottery sherds or vessels were treated by mechanical grinding to obtain a portion of the powdered sample (appropriate for precise weighting and effective extraction). The tool used to obtain ceramic powder was a mini grinder Ryobi ETH150V from Techtronic Industries (Hong Kong, CHN), which ended with the stone tip (silicon carbide). Weighted samples at an amount of 200 mg were placed in Eppendorf-type vials.

In our study, two analytical protocols were utilized due to the requirements of dedicated instrumental techniques. A volume of 10 µL of the internal standard solution was added, where for the GC-MS technique the fentanyl or morphine-D6 for two separate sets of samples. The use of fentanyl as a synthetic internal standard was intentional, as this compound is entirely absent from archaeological matrices and shows no structural similarity to the plant alkaloids targeted in this study, thereby eliminating the risk of analytical interference. Fentanyl provides a stable chromatographic response and consistent ionization under GC-MS/MS conditions, making it a reliable reference for extraction efficiency and signal normalization. This choice follows established toxicological practice, where synthetic standards are employed solely to monitor instrumental performance and procedural stability. The set of samples aimed for native compounds analysis was spiked only with fentanyl as the internal standard and the other set (including the derivatization step) was spiked with morphine-D6. The concentrations of added internal standard solutions were the following, for morphine-D6 500 ng/mL and fentanyl 200 ng/mL, giving final concentrations of respectively 25 ng/g and 10 ng/g in samples. In the next step, 140 µL of ammonium bicarbonate buffer (pH 9) was added and samples were vortex-mixed by Heidolph™ Reax Top mixer (Schwabach, DEU). Then organic solvents such as acetonitrile and ethyl acetate at a volume of 380 µL each were added and again vortex-mixed. After that, the samples were placed in the plastic plate (floated on the water surface) in an ultrasonic bath Sonorex RK 100 H from Bandelin Electronics GmbH (Berlin, DEU) and incubated for 10 min to facilitate the extraction process. Extraction was continued by placing the samples in the automatic shaker Edmund Bühler GmbH SM-30 (Tübingen, DEU) for 5 min at a speed value of 280 moves/minute, and then centrifugation (15,000 rpm/10 min/10 °C) by Sigma 3-16KL centrifuge from SIGMA Laborzentrifugen GmbH (Osterode am Harz, DEU). Obtained supernatants (in the amount of 790 µL) were separated and placed in filtering tubes (equipped with PTFE membrane), and then centrifuged (8800 rpm/2 min/10 °C). Filtered extracts were evaporated to dryness under a gentle stream of nitrogen at 45 °C by the RapidVap Labconco^®^ system (Kansas, MO, USA). Then the one set of evaporated samples (intended for analysis of alkaloids in their native form) was dissolved in 40 µL of methanol and vortexed. The second set of samples (customized for analysis of silylated derivatives of atropine, scopolamine, morphine, and codeine by GC-MS/MS technique) was dissolved in 40 µL of prepared solution (ethyl acetate and Sylon BFT in proportion 1:1), vortexed, and incubated (60 °C for 25 min). All the dissolved samples were centrifuged (14,000 rpm/2 min) and placed in chromatographic vials subjected to instrumental analyses by GC-MS/MS and LC-MS/MS techniques.

## 3. Instrumentation

### 3.1. GC-MS/MS Analysis

Alkaloids in pottery were analyzed in two sets of samples for dedicated GC-MS/MS instrument settings. Our fundamental procedure employed an integrated system of Trace 1310 Gas Chromatograph with TSQ 8000 EVO Triple Quadrupole Mass Spectrometer (Thermo Fisher Scientific^®^, Waltham, MA, USA). The injection was performed with 1 µL of the sample volume in splitless mode, with a 1.2 min valve-off time and a 300 °C temperature maintained. The mobile phase was a constant helium stream at a flow-rate of 1.2 mL/min. For the separation of analytes, the Rxi-5ms fused silica capillary column from Restek (Bellefonte, PA, USA) was used (30 m × 0.25 mm ID × 0.25 µm film thickness), coated with 5% diphenyl/95% dimethyl polysiloxane. The chromatography program was set as follows: from initial 86 °C (0.1 min hold) increased to 230 °C at a rate of 24 °C/min (0.1 min hold) and further elevated to a final value of 314 °C at 15 °C/min (2.2 min hold). The overall analysis time was 14 min. The detection was optimized for electron ionization (EI) and multiple reaction monitoring (MRM) scanning mode. The Auto SRM tool TraceFinder GC (Thermo Fisher Scientific^®^, Waltham, MA, USA) was utilized in the study to establish the favorable detection parameters. Temperatures of detector components were set respectively: 316 °C and 305 °C for the transfer line and the ion source. Spectrometers collision cell was supplied with argon, as the gas inducing ions fragmentation. Optimized ion transitions are summarized in Table 2.

### 3.2. LC-MS/MS Analysis

The other analytical method based on liquid chromatography, was prepared to compare and verify previously obtained results, and to assess the performance and reliability for each of technique. The LC-MS/MS method unlike the one previously described, required only one set of samples (without derivatization step). A device used was a configuration of ultra-high-performance liquid chromatograph Dionex Ultimate 3000 in combination with LTQ Velos Pro—linear ion trap spectrometer (Thermo Fisher Scientific^®^, Waltham, MA, USA). Prepared extracts were sampled at a volume of 10 µL. Analytes were separated on Trace Hypersil Gold column 5 µm, 100 × 3 mm (Thermo Fisher Scientific^®^, Waltham, MA, USA), at a mobile phase flow rate of 0.4 mL/min. Chromatography separation was set as follows: started with 95% B (25 mM ammonium formate, pH 4.5, in water) and 5% A (acetonitrile) and held for 0.8 min, then gradient changed to 70% A and 30% B to analysis time of 12.8 min, followed by a ramp to 100% A and 0% B until the time of 14 min and held to 15.6 min, and finally returned to starting conditions to 16.8 min, and held to endpoint (19.6 min). The elutions from the chromatographic column entered a spectrometer source and ionized in atmospheric pressure chemical ionization (APCI) positive mode, with capillary voltage of 25 V, discharge current of 5 µA, temperatures of 450 °C for the APCI probe, and 180 °C for capillary. Nitrogen streams in ion source were set as follows: sheath gas at 50 arb., and the auxiliary gas at 5 arb. The ion trap spectrometer recorded data in two scanning modes for separate sample runs, the FullMS with a mass range of 50–650 *m*/*z* and then MS-MS (by using helium as collision gas) with energy 35 V of CID (collision-induced dissociation). Data acquisition and processing were carried out with XCalibur software (Thermo Fisher Scientific^®^, Waltham, MA, USA, version 4.3).

## 4. Validation

Chromatographic methods were validated in consideration of the key parameters for bioanalytical assays, including selectivity, linearity, sensitivity, accuracy, precision, recovery, and matrix effect. Selectivity was assessed by the reference samples (n = 10) testing to verify and exclude the potential matrix interferences in retention times (Rt) corresponding to analytes and internal standards. The linearity values for each of the determinations were calculated from prepared five-point calibration curves and expressed as linear regression. The sensitivity parameters as the limit of detection (LOD) and lower limit of quantitation (LLOQ) were calculated with the following equations: LOD = 3σ/S and LLOQ = 10 σ/S, where σ = the standard deviation of concentration at the minimal detector response (n = 10), S = calibration curve slope. Alkaloid recovery for each method was estimated by comparing the area ratio (analyte to internal standard) of four quality control (QC) samples (n = 3 at each concentration) at LLOQ, low (LQC), mid (MQC), and high (HQC) concentrations with recovery samples, prepared from fresh pottery extracts, spiked with appropriate QC standard. The matrix effect was calculated by comparing the area ratio (analyte to IS) between recovery samples and standard solutions at the four QC concentrations prepared in triplicate. The accuracy and precision were determined by the analysis of different QC standards in a triplicate on three consecutive days. Stability evaluation was omitted due to its low importance for the study, where involved materials resided in adverse conditions (difficult to simulate).

## 5. Results

### 5.1. Alkaloids Isolation from Pottery

The final analytical protocol was established as a result of numerous tests involving various component factors of the isolation process such as pH, type of organic solvents, ultrasonic waves, sample purification by centrifugation or filtration, reagents proportions, analytes stability under gas chromatography-mass spectrometry (GC-MS) conditions, derivatization process. Optimal parameters were selected as favorable in terms of the recovery efficacy and versatility in relation to the different compounds targeted.

### 5.2. Validation Results

The demonstrated study emphasized the quality aspect of determinations, thus parameters determining selectivity and sensitivity were considered as the key for our interpretation purposes. Calibrations were made for the calculation of the methods’ analytical limits and working ranges. Quantification of detected substances in actual samples was skipped due to uneven distribution of residues in archeological pottery, and the resulting therefrom difficulties in repeatable and precise concentrations measurement. Validated gas chromatography-mass spectrometry (GC-MS) and liquid chromatography-mass spectrometry (LC-MS) methods demonstrated satisfying selectivity due to lack of interfering peaks in reference samples and high repeatability of retention times and detector responses over-performed runs, in the case of each analyte in artificial QC samples spiked with proper standards. Calibrations included five points from LLOQ to 200 ng/g for the GC-MS/MS method and from LLOQ to 500 ng/g for the LC-MS/MS method. The linear regression (R^2^) of all prepared calibration curves for both methods showed acceptable values of at least R^2^ = 98%.

The variation coefficients (CV%) for precision determinations didn’t exceed 15%, while for accuracy were below 15% in the intra-assay analysis and 20% in the inter-assay analyses. Demonstrated methods present considerable sensitivity differences due to LOD and LLOQ values among them. Whilst, the GC-MS/MS method achieved more favorable results (all LOD and LLOQ values were below 0.5 ng/g). In turn, the LC-MS/MS method showed LOD values ranging from 1–8 ng/g, and the LLOQ in the range of 3–24 ng/g, depending on the analyte type). The set of results for the sensitivity are summarized in Table 3.

The study determining recovery presents slight differences between assays, reaching mean values of 55% for GC-MS, and 61% for LC-MS, respectively. Higher discrepancies in the results occurred between individual analytes, ranging from ±12.7, CV% = 23.1 for GC-MS and respectively ±12.0, CV% = 19.6 for the LC-MS technique. The matrix effects were high for both assays, probably due to applied liquid extraction, where some of the artifact substances remain unseparated until the final step, and then affect the mass spectrometer’s response (by facilitating positive ionization). Discussed recovery and matrix effect parameters are presented in Table 4.

### 5.3. Determination of Alkaloids in Actual Pottery Samples

Samples of each artifact were prepared in duplicate for two sets. The set of samples designed for native compound determination was at first analyzed using the GC-MS method and then the same set of vials was transferred to the LC-MS apparatus and subjected for another analysis. Differently, the sample set of derivatized compounds was analyzed, only by the GC-MS instrument.

#### 5.3.1. GC-MS/MS Instrumental Analysis

**Figure 5:**
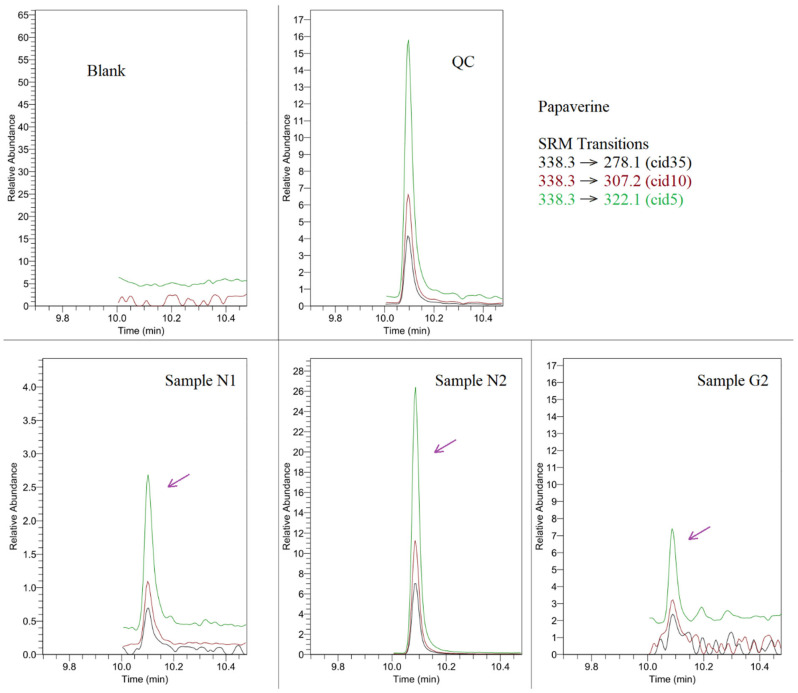
GC-MS/MS overlay chromatograms for papaverine detection window, including blank, QC (at analyte concentration of 4 ng/g), and studied samples N1, N2, and G2.

After GC-MS/MS analysis of native compounds, we recognized papaverine peaks in three of the studied objects (samples N1, N2, G2), which were above the established detection limit, and all had the MRM spectrum consistent with control (QC), as demonstrated in Figure 5.

**Figure 6:**
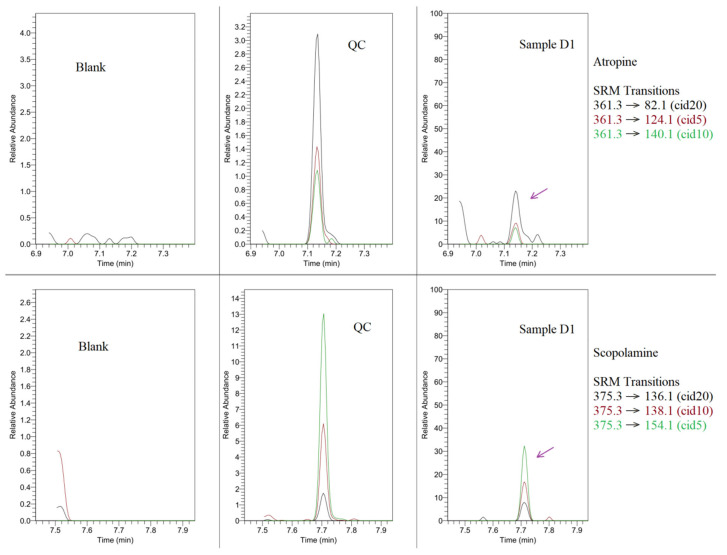
GC-MS/MS overlay chromatograms for atropine (**upper** set) and scopolamine (**bottom** set) detection windows, including blanks, QCs (at analyte concentration of 4 ng/g), and studied sample of D1.

Another analysis performed on the derivatized samples showed in the one of objects (sample D1) the slight peaks corresponding to atropine (being below the established detection limit), and scopolamine in the detectable range (>LOD), but all had appropriate retention times and MRM spectrums in relation to the control (QC). Thus, we assume the presence of these substances as presumable. The argument supporting the credibility of this observation is the fact of the coexistence of those alkaloids (also with other tropane derivatives) in certain *Solanaceae* species, for example, *Atropa belladonna* [8]. The obtained chromatographic results are presented in Figure 6.

#### 5.3.2. LC-MS/MS Instrumental Analysis

**Figure 7:**
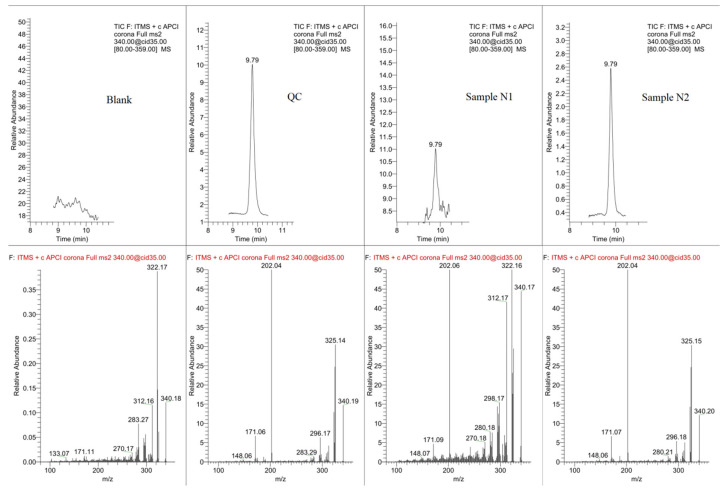
LC-MS/MS chromatograms with integrated MS/MS spectrums for papaverine detection window, including blank, QC (at analyte concentration of 4 ng/g), and studied samples of N1, N2, and G2 [13].

The reference method implemented, confirmed two of the previously obtained results, indicating the papaverine presence in samples N1 and N2. Another sample (G2), which was positive for papaverine in the GC-MS study, didn’t show the corresponding peak of this substance in the LC-MS analysis (in the method’s detection range). The lack of expected papaverine peak in the G2 sample could be argued by differences in sensitivity between the two instrumental methods (where GC-MS appeared to be more sensitive). The obtained positive results are presented in Figure 7, and the identification of papaverine was based on Rt = 9.79 and following MS/MS fragmentation 340 → 202, 325, 171 (where the most intensive product-ion was with mass-to-charge ratio 202). The internal standard (fentanyl) was in turn recognized by the mass-to-charge ratio (*m*/*z*) = 337 and the retention time Rt = 9.92.

The remaining objects as D2, D3, D4, D5, D6, G1 and G3 did not show by any method the presence of targeted substances under established rules of identification. Negative results corresponded by following criteria: to the absence of a detectable signal (peak) on a chromatogram within detection window, a signal below the detection limit for a given substance (concentration calculated by peak area in ng/g), or inconsistencies occurred between the acquired and reference mass spectra that prevented confident identification. Quantitative results from individual measurements were not presented due to significant limitations resulting from the samples properties limiting accuracy, mainly differences in recovery and the phenomenon of uneven distribution of a substance in pottery structure. Therefore, the focus of the study was on the qualitative aspect to reliably detect of a given substance in the analyzed material. The obtained determination results for all studied vessels are summarized in Table 5.

## 6. Discussion

Contemporary archeology has extensive knowledge about the basic areas of life of ancient communities in a specific time and space. This knowledge relates mainly to material culture, e.g., methods of obtaining and producing food, technical skills, acquisition and craft processing of raw materials, and other aspects. To a lesser extent, it refers to ideological content, the material side of which is revealed by cemeteries, as well as to the organizational systems of ancient societies. Thus, prehistoric archaeological finds still hide many mysteries that are difficult to solve, including in the field of medical practices or the use of toxins in antiquity.

Nowak et al. (2020) reported that funnel-beaker people tend to cultivate such plant species as *Triticum dicoccon*, *Triticum monococcum*, or *Hordeum vulgare*, but also *Pisum sativum*, *Lens culinaris*, *Linum usitatissimum*, or *Papaver somniferum* [14]. Studies concerning the absorbed residue were applied for decades with regard to subsistence research. King et al. (2018) performed a similar study to ours using the ultra-high-performance liquid chromatography (UPLC)/tandem mass spectrometry (MS-MS) analysis searching for the presence of atropine in 31 Mississippian ceramic vessels which in fact turned out to be positive for atropine and *Datura* presence [9]. Ceramic evidence of the prehistoric of such species as *Datura* was also presented in other studies concerning e.g., the cultures living in Mexico and the Southwestern United States [15]. Similar investigations were performed in China, where McGovern et al. (2018) using five analytical methods (GC-MS, FT-IR, HPLC-MS, selective Feigl spot tests, as well as stable isotope analysis) revealed the presence of substances with a probable medical significance [16]. Apart from the pottery, the presence of various substances might be detected using hair samples as well; Guerra-Doce et al. (2023) detected the alkaloid epinephrine, atropine, and scopolamine in human hair identifying the usage of multiple drugs by people living in Bronze Age Menorca (Western Mediterranean) [17]. Except for tropane alkaloids, the analyses show that inhabitants could also be exposed to heavy metals along with glycoalkaloids (α-solanine and α-chaconine) [18]. Opium residue was also found in 3500-year-old pottery pieces indicating that this drug could have been used in ancient burial rituals [19]. The other study evidencing prehistorical usage of opium was performed by Vincenti et. al. 2023, where 10 items derived from ancient Daunia in Italy (dating back to the 8th-4th centuries BC) turned out positive for opiates, based on sensitive analyses with UHPLC-tandem mass spectrometry [5].

In our study, we decided to prepare and implement an analytical procedure targeted at alkaloid determination in clay vessels. The assumption related to the prehistoric use of poisonous or hallucinogenic or even medical plants seems to be plausible due to their widespread in the environment as today as in the olden times, dating back to antiquity. Taking into consideration spatial and temporal conditions, we built the hypothesis of prehistoric use of psychoactive plant species representing *Papaverinae* spp. or *Solanaceae* spp. families. Today many natural, physiologically active substances, including the alkaloids of, for instance, opiates (papaverine, morphine, codeine) or tropane derivatives (atropine, scopolamine, cocaine), are applied for medical purposes. Some of them (e.g., hemp, opium poppy, nightshades) have a long history of anthropogenic use, running centuries or even millennia [3,8,11]. Based on the literature, direct and indirect evidence for the psychoactive sources uses have been hitherto discovered, whereas a variety of archeometric instruments and protocols were utilized. This fact hinders the assessment of their reliability through the limited reference abilities [7,8,9,10]. We studied previous works to orient our study, develop the methods, and further compare our results with other papers. In order to make sure about the presence or lack of alkaloids in particular pottery we decided to utilize robust GC-MS/MS and LC-MS/MS instruments, posed the preferred tools in multiple study areas, including pharmaceutical, food testing, environmental, medical, or forensic studies. Our approach is innovative in the archeological field, due to the application of two acknowledged techniques for study. The demonstrated protocol provides a means for the effective isolation of alkaloids deposited on or absorbed within a ceramic fabric, along with their sensitive detection. It holds, that organic residues are demanding tasks for chemical analyses, due to their susceptibility, dependent on time and conditions of residence. Another factor that substantially confounds analytical attempts is the occurrence of strong interactions between organic molecules and pottery matrix particles [7]. The absorption process may be durable, which limits the efficiency of extractions. Therefore, recoveries obtained in our study maximally reached the values of approximately 55–61%. Despite those limitations, our results provide support that methods were effective in detecting traces of organic chemicals in such demanding objects. Besides, we highlighted the insights into the advantages and disadvantages of each of the methods, by assessing their performance in various validation categories. The achievement of more beneficial determination parameters by the GC-MS/MS method prompted us to select it as a fundamental technique, while the other, the LC-MS/MS method was considered as a comparative, reference tool. According to the results of the separate method, which convergingly reflect the presence of papaverine in two specimens (samples N1 and N2), we can be objective at evidencing the purpose of a particular vessel. One of the most important observations accompanying our opiate determination is the lack of morphine and codeine peaks in all the samples positive for papaverine. We verified similar studies and our results were well consistent with Samorini (2019), justifying this phenomenon by stability differences within opiates, where papaverine and thebaine were shown among the most durable. Therefore, it is suggested to evaluate the candidates durability and decomposition phenomenon to select stable alkaloids or their derivatives (such as cotarnine, hydrocotarnine, meconic acid, opianic acid, usually formed from noscapine) [10,11]. The other group of alkaloids tropanes, seems to be also noteworthy according to our observations. Detection of scopolamine and (probably) atropine by our GC-MS assay, requires paying attention to the phenomenon of their occurrence in pottery. The trueness of tropanes presence in our pottery sample (D1) would be surely verified, by utilizing a more sensitive instrument. We can then, be certain to recommend the double-assay protocol in the archeology area, as it introduces a broader view of the study, increasing its versatility and reliability.

## 7. Conclusions

The presented study demonstrated that advanced chromatographic–mass spectrometric techniques (GC-MS/MS and LC-MS/MS) are effective tools for the detection of trace amounts of alkaloids preserved in prehistoric ceramic materials. The applied extraction and derivatization procedures enabled the recovery of structurally diverse compounds despite their millennia-long residence in a porous ceramic matrix. The differences in sensitivity and selectivity between GC-MS/MS and LC-MS/MS highlight the importance of a dual-analytical approach when addressing chemically unstable biomarkers in archaeological materials.

Our results indicate that specific psychoactive alkaloids, such as papaverine and tropane derivatives, can remain detectable in Funnel Beaker Culture vessels, supporting the hypothesis of their non-culinary use in ritual or medicinal contexts. From a materials-science perspective, the study emphasizes the role of ceramic porosity and mineral composition in the preservation, adsorption, and subsequent recovery of organic residues. These findings illustrate the need to integrate material characterization with chemical analysis to better understand long-term stability and degradation pathways of bioactive compounds embedded in ceramics [4].

The developed protocol, validated in terms of recovery, matrix effect, and sensitivity, provides a methodological framework applicable not only to archaeological artifacts but also to broader research on the interaction of bioactive molecules with porous ceramic materials. Such an approach can be extended to heritage science, forensic material studies, and the design of novel diagnostic strategies for ancient residues. Future research should address the influence of microstructural ceramic properties on biomolecule retention and explore hybrid analytical workflows combining spectroscopic and chromatographic techniques to enhance detection reliability.

## Figures and Tables

**Table 1:** Names and characteristics of individual FBC pottery from studied assemblage.

Sample Name	Pottery Information	
	Location	Affiliation
D1	Dubeczno 1, Włodawa district	miniature
D2		miniature
D3		miniature
D4		miniature
D5		miniature
D6		miniature
G1	Gródek 1C, Hrubieszów district	miniature
G2		miniature
G3		miniature
N1	Nałęczów Plateau, Puławy district	collared flask
N2	Zgórzyńskie, Puławy district	collared flask
W1	Wąwolnica 7, Puławy district	collared flask

**Table 2:** Detection parameters of alkaloids for GC-MS/MS method, with listed sample sets.

Sample Set of Silylated Compounds					
Name	Rt	Mass	Product Mass	Collision Energy	Ion Polarity
Atropine-TMS	7.13	361.3	82.1	20	Positive
			124.1	5	
			140.1	10	
Scopolamine-TMS	7.71	375.3	136.1	20	
			138.1	10	
			154.1	5	
Codeine-TMS	8.22	234.2	162.1	20	
		371.3	146.1	25	
			214.1	30	
Morphine-D6-diTMS (IS)	8.44	239.1	149.1	10	
		435.2	293.1	15	
Morphine-diTMS	8.46	236.2	146.1	10	
		429.3	234.2	10	
			401.2	10	
Sample Set of Native Compounds					
Name	Rt	Mass	Product Mass	Collision Energy	Ion Polarity
Fentanyl (IS)	9.55	245.2	146.1	15	Positive
			189.2	10	
Papaverine	10.12	338.3	278.1	35	
			307.2	10	
			322.1	15	

**Table 3:** Sensitivity parameters of LOD and LLOQ for developed analytical methods.

Compound	Analytical Limits (ng/g)			
	GC-MS/MS		LC-MS/MS	
	LOD	LLOQ	LOD	LLOQ
Atropine	0.1	0.3	1	3
Scopolamine	0.04	0.12	8	24
Morphine	0.04	0.12	5	15
Codeine	0.04	0.12	5	15
Papaverine	0.1	0.3	1	3

**Table 4:** Recoveries and matrix effects for established analytical methods.

Compound	% Recovery		% Matrix Effect	
	GC-MS/MS	LC-MS/MS	GC-MS/MS	LC-MS/MS
Atropine	54.4	63.5	103.4	158.4
Scopolamine	47.2	63.8	133.6	153.6
Morphine	50.0	57.8	240.8	375.2
Codeine	45.9	44.4	249.6	253.1
Papaverine	76.8	77.7	207.8	137.1
Average	54.9	61.4	187.0	215.5

**Table 5:** Summarizing table of analytical results on real samples.

Sample Name	Analytical Results-Compounds Detected by Method	
	GC-MS/MS	LC-MS/MS
D1	negative but probable-atropine (not confirmed by result below LOD), positive-scopolamine	negative
D2	negative	negative
D3	negative	negative
D4	negative	negative
D5	negative	negative
D6	negative	negative
G1	negative	negative
G2	positive-papaverine	negative
G3	negative	negative
N1	positive-papaverine	positive-papaverine
N2	positive-papaverine	positive-papaverine
W1	negative	negative

## Data Availability

The original contributions presented in the study are included in the article, further inquiries can be directed to the corresponding authors.

## Abbreviations

APCI	Atmospheric Pressure Chemical Ionization
BSTFA	N,O-Bis(trimethylsilyl)trifluoroacetamide
CID	Collision-Induced Dissociation
CV	Coefficient of Variation
EI	Electron Ionization
FBC	Funnel Beaker Culture
FT-IR	Fourier Transform Infrared Spectroscopy
GC-MS	Gas Chromatography–Mass Spectrometry
GC-MS/MS	Gas Chromatography–Tandem Mass Spectrometry
HPLC-MS	High-Performance Liquid Chromatography–Mass Spectrometry
HQC	High-Level Quality Control
ID	Inner Diameter
IS	Internal Standard
LC-MS	Liquid Chromatography–Mass Spectrometry
LC-MS/MS	Liquid Chromatography–Tandem Mass Spectrometry
LLOQ	Lower Limit of Quantitation
LOD	Limit of Detection
LQC	Low-Level Quality Control
MQC	Mid-Level Quality Control
MRM	Multiple Reaction Monitoring
MS	Mass Spectrometry
MS/MS	Tandem Mass Spectrometry
*m*/*z*	Mass-to-Charge Ratio
PTFE	Polytetrafluoroethylene
QC	Quality Control
Rt	Retention Time
SRM	Selected Reaction Monitoring
TMCS	Trimethylchlorosilane
UHPLC	Ultra-High-Performance Liquid Chromatography
